# Pyk2/MCU Pathway as a New Target for Reversing Atherosclerosis

**DOI:** 10.3389/fcell.2021.651579

**Published:** 2021-05-06

**Authors:** Yingzhen Zhang, Xiaoli Yang, Zhongzhong Li, Kailin Bu, Tong Li, Zhizhao Ma, Binbin Wang, Lina Ma, Honglin Lu, Kun Zhang, Luji Liu, Yanying Zhao, Yipu Zhu, Jin Qin, Junzhao Cui, Lin Liu, Shuxia Liu, Ping Fan, Xiaoyun Liu

**Affiliations:** ^1^Department of Neurology, The Second Hospital of Hebei Medical University, Shijiazhuang, China; ^2^Department of Basic Medicine, Hebei Medical University, Shijiazhuang, China; ^3^Affiliated Hospital of Hebei University of Engineering, Handan, China; ^4^Neurosurgery Department, The Second Hospital of Hebei Medical University, Shijiazhuang, China; ^5^Neuroscience Research Center, Medicine and Health Institute, Hebei Medical University, Shijiazhuang, China

**Keywords:** atherosclerosis, mitochondrion, Pyk2/MCU, ApoE^–/–^ mice, HUVECs

## Abstract

**Objective:** Multiple mechanisms including vascular endothelial cell damage have a critical role in the formation and development of atherosclerosis (AS), but the specific molecular mechanisms are not exactly clarified. This study aims to determine the possible roles of proline-rich tyrosine kinase 2 (Pyk2)/mitochondrial calcium uniporter (MCU) pathway in AS mouse model and H_2_O_2_-induced endothelial cell damage model and explore its possible mechanisms.

**Approach and Results:** The AS mouse model was established using apolipoprotein E-knockout (ApoE^–/–^) mice that were fed with a high-fat diet. It was very interesting to find that Pyk2/MCU expression was significantly increased in the artery wall of atherosclerotic mice and human umbilical vein endothelial cells (HUVECs) attacked by hydrogen peroxide (H_2_O_2_). In addition, down-regulation of Pyk2 by short hairpin RNA (shRNA) protected HUVECs from H_2_O_2_ insult. Furthermore, treatment with rosuvastatin on AS mouse model and H_2_O_2_-induced HUVEC injury model showed a protective effect against AS by inhibiting the Pyk2/MCU pathway, which maintained calcium balance, prevented the mitochondrial damage and reactive oxygen species production, and eventually inhibited cell apoptosis.

**Conclusion:** Our results provide important insight into the initiation of the Pyk2/MCU pathway involved in AS-related endothelial cell damage, which may be a new promising target for atherosclerosis intervention.

## Highlights

- Pyk2/MCU regulates mitochondrial Ca^2 +^ uptake, ROS production, mitochondrial membrane potential and apoptotic signaling, which affects the occurrence and development of atherosclerosis at a certain extent.
- Down-regulation of Pyk2 by short hairpin RNA (shRNA) protected HUVECs from H_2_O_2_ insult.
- Pyk2/MCU pathway can be inhibited by the treatment of rosuvastatin which showed a protective effect on AS mouse model and H_2_O_2_-induced HUVECs injury.

## Introduction

Atherosclerosis (AS) is the leading cause of cardiovascular and cerebrovascular disorders such as myocardial infarction and brain stroke (Huang et al., 2019). Dysregulation of lipid metabolism and inflammatory processes that involve the binding of monocytes to dysfunctional endothelium, their recruitment to susceptible areas of the arterial wall, and their differentiation to macrophages and development into foam cells have been associated with atherosclerosis formation (Yu et al., 2018). Several other pathogeneses of atherosclerosis have been suggested, including lipid infiltration theory, thrombosis theory, and smooth muscle cell cloning theory. The theory of endothelial injury response is recently reported as a major factor for this disease, which renders the modulation of endothelial cell (EC) functions a key therapeutic target (Widlansky and Gutterman, 2011; Sandow et al., 2012; Godo and Shimokawa, 2017). The change of intracellular Ca^2+^ concentration ([Ca^2+^]_i_) has an important role in ECs’ functions (Alevriadou et al., 2017). In most cells, fluctuations in Ca^2+^ concentration are translated into the production of cellular signals, while [Ca^2+^]_i_ transients are defined and shaped by the mitochondria. Ca^2+^ homeostasis regulates numerous cell functions, including energy metabolism, reactive oxygen species (ROS) generation, spatiotemporal dynamics of Ca^2+^ signaling, cell growth, and cell death. The mitochondria are highly specialized in this context because of their sponge-like retentive capacity with calcium balance (Smith and Gallo, 2018).

The cell has a complicated mitochondrial Ca^2+^ transport mechanism that controls Ca^2+^ entry to the mitochondrial matrix and exit to the cytosol. Mitochondria have a large negative membrane potential which may reach 180 mV, which facilitates flooding of mitochondria Ca^2+^ from the cytoplasm (Mallilankaraman et al., 2012). On the other hand, the heterogeneous protein complex with mixed molecular identity assembled into mitochondrial calcium uniporter (MCU) is a new core component that is identified in 2011, which regulates the influx of calcium ions into the mitochondria (Baughman et al., 2011). Previous studies have found that MCU expression regulates the buffering of cytoplasmic Ca^2+^ during systole in neonatal rat cardiomyocytes (Drago et al., 2012). In pancreatic β cells, MCU regulates cellular glucose sensing capacity (Tarasov et al., 2012). Thus, MCU plays a specific role in the occurrence and development of diseases. However, how MCU activity contributes to EC dysfunction has received considerably less attention. Non-receptor calcium-dependent proline-rich tyrosine kinase 2 (Pyk2) is an important signaling molecule that senses changes of intracellular calcium levels and induces alterations in cell function (Hirschler-Laszkiewicz et al., 2018). Recently, studies have reported that Pyk2 regulates mitochondrial calcium uptake through phosphorylation of the MCU in myocardial cells (O-Uchi et al., 2014). In addition, in our previous study, we did find that the Pyk2/MCU pathway was activated in a rat cerebral ischemia model, which was responsible for mitochondrial dysfunction, calcium balance, and neuronal apoptosis (Zhang et al., 2018). Meanwhile, by reviewing the literature, it was reported that angiotensin II (Ang II) stimulated a calcium-sensitive tyrosine kinase Pyk2 *via* the type-1 angiotensin II (AT1) receptor in pulmonary vein endothelial cells (PVECs) and vascular smooth muscle cells (VSMCs) (Tang et al., 2000; Satoh et al., 2001; Nakashima et al., 2006). In the endothelial cells, Ang II binds to EC receptors and facilitates the expression of plasminogen activator inhibitor-1 and cell adhesion molecules, which may promote the development of atherosclerosis and myocardial infarction (Luscinskas and Gimbrone, 1996; St Paul et al., 2020), due to promoting ROS generation, increasing the cell-surface expression of cell adhesion molecules, and enhancing leukocyte adhesiveness to ECs, eventually causing endothelium dysfunction. Would the presence of Ang II and EC injury in the initial stage of atherosclerosis activate the Pyk2/MCU pathway (further aggravating the progression of the disease)? We would address the above-mentioned questions in this study.

Therefore, we observed the changes of the Pyk2/MCU pathway in the H_2_O_2_-induced endothelial cell injury model and the AS mouse model, and we used rosuvastatin (Rosu) to treat the EC injury model and AS mouse model. Statins have long been used to lower cholesterol levels through inhibition of 3-hydroxy-3-methylglutaryl coenzyme A (HMG-CoA) reductase, the rate-limiting enzyme in the mevalonate pathway (Lozano-Cuenca et al., 2020). Recently, their anti-inflammatory and endothelial cells’ protective actions have been proposed independently of their anti-hyperlipidemic effects (Escudero et al., 2015). Meantime, evidence had shown that statins could block Ang II-induced Pyk2 activation in PVECs (Satoh et al., 2001), which provided an idea for us to study the molecular pathways related to Pyk2 and the therapeutic mechanism of rosuvastatin. In the present study, it will be observed if rosuvastatin could inhibit Ang II and the Pyk2/MCU pathway simultaneously in the development process of atherosclerosis, which would further identify the Pyk2/MCU pathway as a new target to prevent EC dysfunction and AS progression as well as Ang II related to Pyk2/MCU at some levels.

## Materials and Methods

### Animals

Male apolipoprotein E-knockout (ApoE^–/–^) mice (B6.129P2-Apoe^*tm1Unc*^/J) were a kind gift of Professor Zheng Bin (Biochemistry Department of Hebei Medical University, Shijiazhuang, China). Male C57BL/6J mice (6 weeks old) were purchased from Beijing Vital River Laboratory Animal Technology Co., Ltd. (China). All the animals were housed in an environment with temperature of 22 ± 1°C, relative humidity of 50 ± 1%, and a light/dark cycle of 12/12 h. At 8 weeks of age, randomized ApoE^–/–^ mice and C57BL/6J mice were fed with a high-fat diet (Beijing Keao Xieli Feed Co., Ltd., China) containing 78.85% basic feed, 21% lard, and 0.15% cholesterol, while the control groups (ApoE^–/–^ mice and C57BL/6J mice) were given a general chow diet. Furthermore, all *in vivo* experiments (including the mice euthanasia procedure) were conducted in accordance with the requirements and principles of the Animal Care and Use Committee of Hebei Medical University as well as the AAALAC and the IACUC guidelines.

### Animal Model of Atherosclerosis

An atherosclerotic mouse model was induced in male ApoE^–/–^ mice that were fed with a high-fat diet for 12 weeks. After 12 weeks, three mice were randomly selected to examine the formation of plaque in artery. All the mice were divided into five groups: C57BL/6J+general diet (*n* = 11), ApoE^–/–^+general diet (*n* = 11), C57BL/6J+high-fat diet (*n* = 13), ApoE^–/–^+high-fat diet (*n* = 13), and ApoE^–/–^+high-fat diet+Rosu (*n* = 13). After 12 weeks, the drug treatment group (ApoE^–/–^+high-fat diet+Rosu) received an intragastric administration of 5 mg/kg/day rosuvastatin (MedChemExpress, United States) according to the previous protocol (Calkin et al., 2008) for 4 weeks, while other mice received the same amount of saline solution. After 4 weeks, all the mice were anesthetized with 10% hydrated chlorine aldehyde.

### Cell Culture and Treatments

HUVECs were obtained from the BeNa Culture Collection (Beijing, China). The cells were cultured in Dulbecco’s modified Eagle’s Medium (DMEM) supplemented with 10% fetal bovine serum (Gibco, United States), streptomycin (100 μg/ml), and penicillin (100 IU/ml) in a humidified atmosphere containing 5% CO_2_/95% air at 37°C. The culture medium was replaced every 2 days.

In the experiment, HUVECs were pretreated with rosuvastatin (MedChemExpress, United States; 2.5, 5, and 10 μM) for 2 h. The concentrations were determined prior to the experiment with a drug concentration gradient (Wang et al., 2010). Rosuvastatin was dissolved at a certain concentration in dimethyl sulfoxide (DMSO) (Piconi et al., 2008); the final concentration of DMSO was always lower than 0.01%, which had been shown to have no effect on cell viability (Gao et al., 2008). The cells were then exposed to H_2_O_2_ (750 μM) for 24 h (Chen et al., 2014). The final concentrations of H_2_O_2_ were determined by the experiment with a H_2_O_2_ concentration gradient.

### Cell Viability Assay

Cell viability was measured with the CellTiter 96^®^ AQueous One Solution Cell Proliferation Assay (Promega Corporation, United States). After 24 h exposure to H_2_O_2_, 20 μl of MTS [3-(4,5-dimethylthiazol-2-yl)-5-(3-carboxymethoxyphenyl)-2-(4-sulfophenyl)-2H-tetrazolium, inner salt] was added to each well and the plates were incubated at 37°C for 4 h. After removal of the medium, 100 μl DMEM was added to each well to avoid previous additions affecting absorbance values. Absorbance was measured at 490 nm by a microplate reader (Bioteck, Inc., Winooski, VT, United States).

### Serum Lipid and Angiotensin II Detection

After 16 weeks of intervention, all mice were fasted overnight. Blood was collected from retro-bulbar sinus. Blood samples were then centrifuged at 2500 rpm for 5 min, and the serum and the plasma were collected, respectively. Concretely, total cholesterol (TC), triglyceride (TG), high-density lipoprotein cholesterol (HDL-c), low-density lipoprotein cholesterol (LDL-c) of serum, and Ang II of plasma were detected (Yi He Medical Laboratory, Shijiazhuang, China), respectively. The concentrations of Ang II in conditioned HUVECs culture media were measured using human angiopoietin-2 ELISA Kit (MultiSciences, China) according to the manufacturer’s instructions. In brief, cells were collected, washed in phosphate-buffered saline (PBS) three times, and then resuspended. The cells were then shocked and fragmented using Ultrasonic Cell Disruptor. The cell suspension was centrifuged, and the supernatant was taken to determine the concentration of Ang II.

### Histopathological Analysis

The mice were perfused through the left ventricle with normal saline and then fixed *in situ* with 4% paraformaldehyde. For each mouse, the artery from the aorta root to the iliac artery was dissected, dehydrated overnight in 30% sucrose, the length from the aortic root coming out of the heart to before the bifurcation of the aortic arch embedded in optimum cutting temperature compound, and finally frozen. Continuous frozen sections (5-μm-thick) obtained by a Leica CM3050S frozen slicer (Germany Leica Co., Ltd.) were made from the aortic root to the arch of the aorta before it bifurcated. The frozen sections were stained with Oil red O and hematoxylin–eosin (HE) to analyze plaque relative area and tissue damage, respectively, and the results were observed with an optical microscope (Germany Leica Co., Ltd.). The remaining arterial tissues were stained with Oil red O as observed with a Smz-b4 microscope (Chongqing Otter Company, China).

Moreover, we detected the expression of CD31, Pyk2, and MCU in the artery tissues. The frozen sections were placed in a 37°C electrothermostat for 30 min. The sections were then washed with 0.01 M PBS three times, blocked with goat serum, and placed in a moist chamber at room temperature for 1 h. The sections were then incubated with purified rat anti-mouse CD31 antibody (1:10, BD Pharmingen, United States), anti-MCU polyclonal antibody (1:100, Bioworld Technology, United States), and anti-Pyk2 monoclonal antibody (1:60, Abcam, United States) at 4°C overnight, following incubation with the goat anti-rabbit Dylight 594 fluorescence secondary antibody (1:300, Abbkine, United States) and goat anti-rat IgG, FITC conjugated (1:100, CWBIO, China) for 1 h at room temperature. Finally, DAPI (Boster Biological Technology Co., Ltd., United States) was used to dye the nucleus. The immunofluorescent results were photographed with a BX61 universal microscope (Japan Olympus Co., Ltd.). To detect the protein expression in immunofluorescence images, we counted the mean of integrated option density (IOD) in order to reduce artificial errors. The mean of IOD = (IOD)/SUM AREA of the image that was selected in this experiment, that is, the optical density values of each point in the target aorta AREA on the image were first summed up to obtain IOD and then divided by the AREA of the effective target distribution AREA to obtain the mean density, which reflected the concentration per unit AREA of the target substance. An automated image analysis system (Image-Pro Plus 6.0; Media Cybernetics, Silver Spring, MD, United States) was used for all quantitative measurements.

### Western Blot Analysis

Harvested cells and artery tissues from the aorta root to the iliac artery were used for western blotting. Cold radioimmunoprecipitation assay lysis buffer and phenylmethylsulfonyl fluoride protease inhibitors (Beijing Solarbio Science & Technology Co., Ltd., China) were used for protein extraction following the manufacturer’s protocol. Protein concentration was determined with the BCA Protein Assay Reagent Kit (Beijing Solarbio Science & Technology Co., Ltd., China). Then, 30 μg total proteins were resolved by electrophoresis and then transferred onto polyvinylidene difluoride membranes (Millipore Corporation, United States). After blocking with 5% skim milk for 1 h at room temperature, the membranes were incubated at 4°C overnight with anti-MCU (1:2,000, Cellsignal), anti-Pyk2 (1:1,000, Abcam), and anti-caspase-3 (1:1,000, Signalway, United States/Proteintech, China) antibodies diluted in 5% skim milk. Polyclonal mouse anti-β-actin antibody (1:3,000, Bioworld Technology) and anti-GAPDH polyclonal antibody (1:3,000, Atagenix, Wuhan, China) were used as an internal control. On the next day, the membranes were incubated with fluorescent-labeled secondary antibodies (Dylight 800, goat anti-rabbit IgG, 1:2,000, Abbkine) for 1 h at room temperature; after washing, they were scanned and analyzed on an Odyssey infrared laser scanner (LICOR Bioscience, Lincoln, NE, United States). Densitometric values were normalized to β-actin or GAPDH immunoreactivity to correct for any loading and transfer differences among samples.

### Ultrastructural Observation of Tissues and Cells

Mice were perfused with 0.9% normal saline followed by 4% paraformaldehyde and 4% glutaraldehyde in 0.9% normal saline. The wall of the blood vessel from the aortic root coming out of the heart to before the bifurcation of the aortic arch was cut into 1 × 1-mm pieces and then fixed in 4% glutaraldehyde for 24 h. Next, the samples were post-fixed with 1% osmium tetroxide for 2 h at 4°C. After dehydration with ethyl alcohol, the samples were embedded in Epon^TM^ resin. Ultrathin sections were cut and stained with uranyl acetate and lead citrate. To obtain the cell samples, the HUVECs were digested with 0.25% trypsin, washed with PBS three times and collected in 1.5-ml EP tubes. The cell suspension was then centrifuged at 1,000 rpm for 3 min, and the supernatant was discarded. Cell mass was suspended with 4% glutaraldehyde and fixed for 24 h. The further procedures of dehydration, fixation, sectioning, and staining were the same as those used for the collected mice specimens. The specimens were observed and photographed with a JEM-1230 (Japan) electron microscope.

### Real-Time Quantitative Polymerase Chain Reaction

The total RNA of HUVECs was extracted with Trizol Reagent (Invitrogen, Carlsbad, CA, United States) according to the manufacturer’s instructions. RNA (2 μg) from each sample was converted to cDNA. The primers were synthesized by Beijing Saibaisheng Gene Technology Co., Ltd. The forward and reverse primers that were used were as follows: 5′-AGGATCGGGGAATTGACAGAG-3′ (for ward) and 5′-GTGTGGTGTATAGTTGCTGGAC-3′ (rev erse) for MCU, 5′-GAAGCCGAGTGGAGGTATG-3′ (forw ard) and 5′-GCGCTGCATGTAGTCGTT-3′ (reverse), 5′-GTGGCCGTCAAGACCTGTAA-3′ (forward) and 5′-AGC TTGACGATGTGAGGGTG-3′ (reverse) for Pyk2, 5′-GAGCTA CGAGCTGCCTGAC-3′ (forward) and 5′-GGTAGTTTCGTGG ATGCCACAG-3′ (reverse) for β-actin, and 5′-TCAAG AAGGTGGTGAAGCAGG-3′ (forward) and 5′-TCAA AGGTGGAGGAGTGGGT-3′ (forward) for GAPDH. All samples were normalized to β-actin or GAPDH. The relative gene copy number for each sample was measured using the comparative threshold cycle number (Ct) method. Each sample was processed in triplicate. Relative gene expression was evaluated using the formula 2^–ΔΔCt^ method as previously described (Plengvidhya et al., 2012).

### Mitochondrial Membrane Potential

The HUVECs with different treatments were harvested using 0.25% trypsin without ethylenediaminetetraacetic acid and washed with PBS three times. Finally, we used PBS to resuspend the cells, and Rhodamine 123 (Sigma-Aldrich, Steinheim, Germany) was used to detect the mitochondrial membrane potential (MMP). Rhodamine 123 (2 μM) was added to dissociated cells for 30 min at 37°C, and the cells were then washed three times with PBS. Lastly, fluorescence intensity was measured by flow cytometry (Cytomics FC 500, Beckman Coulter, United States).

### Analysis of Reactive Oxygen Species

The production of ROS was determined by using ROS-sensitive fluorescent probe 2′,7′-dichlorofluorescein diacetate (DCFH-DA) (Beyotime, Shanghai, China), following the manufacturer’s instruction. In brief, the cells were harvested as described above, resuspended in 1 ml PBS with 1 μl DCFH-DA (10 μM), incubated in the dark at 37°C for 20–30 min, and then washed three times with PBS. Fluorescence was then measured with a Cytomics FC 500, Beckman Coulter flow cytometer (United States).

### Intracellular Free Ca^2+^ Concentration

The fluorescent Ca^2+^ indicator Fluo-4/AM([Ca^2+^]_i_) (Invitrogen, United States) was used to detect the intracellular free calcium concentration. The HUVECs were prepared as described above. A total of 2 μM Fluo-4/AM was then added for 30 min at 37°C. The changes in [Ca^2+^]_i_ were measured with a flow cytometer (CytomicsFC500, Beckman Coulter, United States) and expressed as relative fluorescence intensity.

### Cell Apoptosis

The number of apoptotic cells was detected by using Annexin V-FITC Apoptosis Detection Kit (Beyotime, Shanghai, China), following the manufacturer’s instruction. Briefly, HUVECs were collected as previously described. The cells were then resuspended in 200 μl binding buffer, incubated with 5 μl Annexin V-FITC and 10 μl PI, gently mixed, and then placed in the dark at room temperature for 15 min. Finally, the cells were mixed with 300 μl binding buffer and analyzed using flow cytometry (FACSCanto^TM^ II, BD, United States).

### Pyk2 Gene Silencing by shRNA

Four short hairpin RNAs (shRNAs) targeting the Pyk2 gene as well as a scramble shRNA (NC, non-specific control) were both designed and synthesized by the company of GeneCopoeia (GeneCopoeia Inc., United States). Several different shRNAs of mRNA at different target sites were selected, and an effective interference sequence was screened out through the preliminary experiment (Figure 7A). As can be seen from Figure 7A, the infection efficiency of the cells that enhanced green fluorescent protein (EGFP) co-expressed with shRNA in the five groups had reached 80–90%. The interference sequence of shRNA and EGFP was initiated by different promoters: the interference sequence of shRNA was initiated by H1, and EGFP was initiated by SV40. Then, real-time quantitative PCR (rt-qPCR) and western blot were used for further identification to determine the virus that made Pyk2 of low expression better.

HUVECs were normally cultured, and then the slabs were laid with 2.5 × 10^5^ cells/well (six-well plates) and cultured overnight at 37°C. When the cells were in good condition, HUVECs were infected by lentiviral particles according to a different multiplicity of infection (MOI) on the next day, and repeated infection was conducted 8 h later. Fresh culture medium was changed on the third day. At 3 days after transfection, the expression of green fluorescent protein was observed with a fluorescence microscope, and the transfection efficiency (green fluorescent protein-positive cell rate) was calculated. Meanwhile, western blot and rt-qPCR were performed to examine the low expression effect of the gene. Next, HUVECs were divided into several groups: blank group (control), blank+H_2_O_2_ group, NC (non-specific control) +H_2_O_2_ group, shRNA+H_2_O_2_ group, H_2_O_2_+Rosu group, and shRNA+H_2_O_2_+Rosu group. MTS was applied to detect the changes of cell viability in different groups, flow cytometry was used to detect changes in intracellular Ca^2+^ and mitochondrial membrane potential, and western blot was adopted to detect the protein expression of Pyk2, MCU, caspase-3, and GAPDH. The methods had been described above.

### Statistical Analysis

All results were shown as mean ± SD. Statistical comparisons were preformed using one-way ANOVA followed by Student–Newman–Keuls test and least significant difference test for multiple comparisons. Student’s *t*-test and Mann–Whitney nonparametric tests were applied to compare variables between two groups (*a* = 0.05 as inspection level). Results with *P* < 0.05 were considered statistically significant. SPSS 23.0 statistical software (IBM SPSS, Chicago, IL, United States) was used for statistical analysis.

## Results

### Validation of the Atherosclerotic Mouse Model and the Therapeutic Effect of Rosuvastatin on Atherosclerosis

**FIGURE 1:**
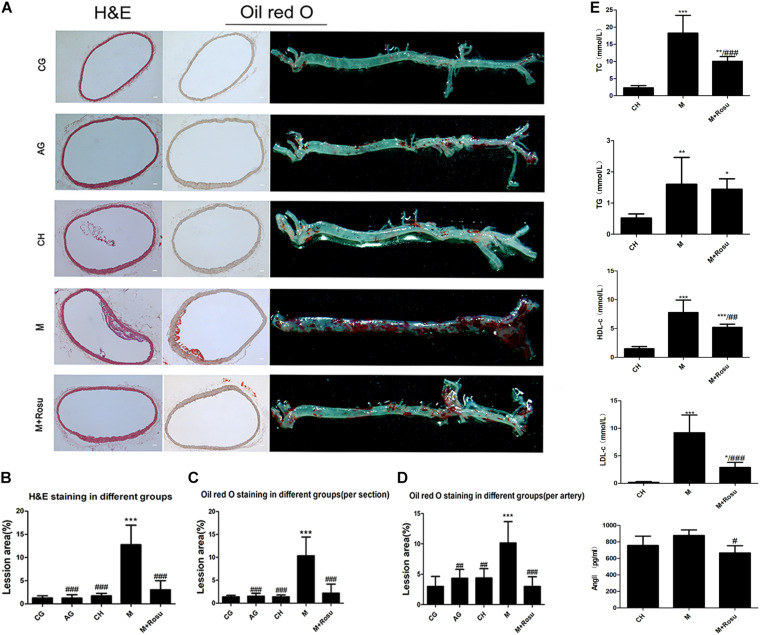
The lesion degree of atherosclerosis and the effect of rosuvastatin in ApoE^– /–^ mice. **(A)** HE pathological section, Oil red O pathological section, gross arterial tissue Oil red O staining. Scale bar: 50 μm. **(B–D)** The relative lesion area of plaques was calculated by Image Pro Plus 6.0. **(E)** Concentration of lipids and Ang II in different animal groups. Data are shown as mean ± SD of at least three independent experiments. *P < 0.05, **P < 0.01, ***P < 0.001 *versus* vehicle CH; ^#^*P* < 0.05, ^##^*P* < 0.01, ^###^*P* < 0.001 *versus* vehicle model (M), one-way ANOVA. CG, C57BL/6J+general diet; AG, ApoE^– /–^ +general diet; CH, C57BL/6J+high-fat diet; M, ApoE^– /–^ +high-fat diet; M+Rosu, ApoE^– /–^ +high-fat diet+Rosu.

Oil red O and HE staining were used to stain the lipid plaque in aortic lesions (Figure 1A). As expected, we found lipid accumulation in the inner wall of the aortic and the relative plaque area, which was much larger and necrotic in the model group (ApoE^–/–^+high-fat diet). On the contrary, the plaque area in the group treated with rosuvastatin was significantly smaller and smoother in different degrees compared to the model group (Figures 1B–D).

Furthermore, the levels of serum lipids were spontaneously elevated in the model group. Compared with the CH (C57BL/6J+high-fat diet) group, the levels of TC, TG, HDL-c, and LDL-c were all increased in the model group (Figure 1E). In addition, those levels were significantly decreased in the rosuvastatin group compared to the model group except the level of TG. On the other hand, compared with the CH group, the level of Ang II in the model group was slightly increased while it was not significant, and the concentration of Ang II (Figure 1E) revealed a significant decline in the rosuvastatin group compared to the model group.

**FIGURE 2:**
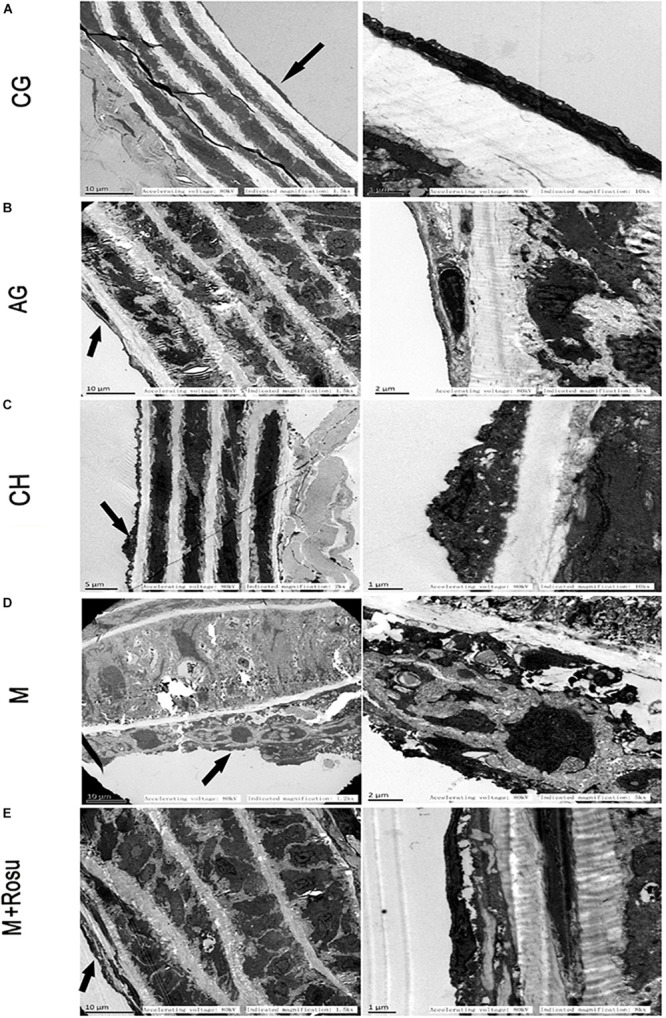
Ultrastructural changes in arterial wall during atherosclerosis. **(A)** C57BL/6J+general diet. The endothelial cells of endodermis had normal nuclear and cytoplasmic contents, vascular wall lamination was clearly visible, and smooth muscle layer thickness was uniform. **(B)** ApoE^– /–^ +general diet. The endothelial cells of endodermis changed slightly; vascular wall lamination was visible, while the smooth muscle layer thickened slightly. **(C)** C57BL/6J+high-fat diet. We can see changes in the morphology of endothelial cells and thickening of the smooth muscle layer. **(D)** ApoE^– /–^ +high-fat diet. Significant morphological transformation was observed in this group, which was characterized by thickening of the smooth muscle layer, endothelial cell shape changes (from long fusiform to round and spherical shape) and nuclear pyknosis, cytoplasmic condensation, and cell volume increasing. **(E)** ApoE^– /–^ +high-fat diet+Rosu. The morphology of the arterial wall in the rosuvastatin group had less damage compared with the model group; the edema of the endothelial cells was mitigated, the organelles’ structure was recovered gradually, and the nucleoli and the nuclear membrane seemed intact. The arrow shows the endothelial cell. The area indicated by the arrow is enlarged in the right column. CG, C57BL/6J+general diet; AG, ApoE^– /–^ +general diet; CH, C57BL/6J+high-fat diet; M, ApoE^– /–^ +high-fat diet; M+Rosu, ApoE^– /–^ +high-fat diet+Rosu.

To observe the ultrastructural changes of vascular wall during atherosclerosis, transmission electron microscopy was used to examine the ultrastructure of the segment from the aortic root coming out of the heart to before the bifurcation of the aortic arch. The endothelial cells of the endodermis from the control group had normal nuclear and cytoplasmic contents, vascular wall lamination was clearly visible, and smooth muscle layer thickness was uniform (Figure 2A). In the AG group (ApoE^–/–^+general diet), we can see that the endothelial cells of endodermis changed slightly and vascular wall lamination was visible, while the smooth muscle layer thickened slightly (Figure 2B). In the CH group (C57BL/6J+high-fat diet), what we can see are the changes in the morphology of endothelial cells and thickening of the smooth muscle layer (Figure 2C). On the other hand, significant morphological transformation was observed in the model group, which was characterized by thickening of the smooth muscle lay, endothelial cell shape changes (from long fusiform to round and spherical shape) and nuclear pyknosis, cytoplasmic condensation, and cell volume increase (Figure 2D). In addition, the endothelial cells had obvious edema, membrane dissolution, severe intracytoplasmic vacuolization, rare intact organelles, nuclear membrane dissolution, nuclear chromatin edge set, cell body deformation, and nuclear condensation. On the contrary, the morphology of the arterial wall in the rosuvastatin group had less damage compared with the model group; the edema of the endothelial cells was mitigated, the organelles’ structure was recovered almost similarly to the normal, the nucleoli and the nuclear membrane seemed intact (Figure 2E).

### The Expression of Pyk2/MCU in the Artery Wall Underlying Atherosclerosis

**FIGURE 3:**
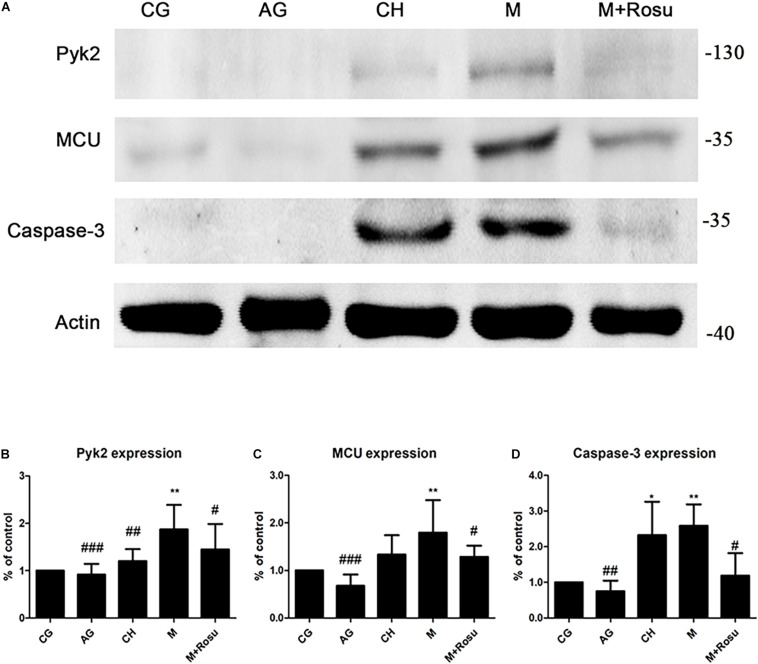
The expression of Pyk2/mitochondrial calcium uniporter (MCU) after atherosclerosis (AS) and rosuvastatin intervention in a mouse model of AS by western blot. **(A)** The protein expression of Pyk2, MCU, and caspase-3. **(B–D)** Statistical graph of Pyk2 **(B)**, MCU **(C)**, and caspase-3 **(D)**. Data are shown as mean ± SD of at least three independent experiments. *P < 0.05, **P < 0.01 *versus* vehicle control (CG); ^#^*P* < 0.05, ^##^*P* < 0.01, ^###^*P* < 0.001 versus vehicle model (M), one-way ANOVA and unpaired Student’s *t*-test (*a* = 0.05 as inspection level, M *versus* M+Rosu). CG, C57BL/6J+general diet; AG, ApoE^– /–^ +general diet; CH, C57BL/6J+high-fat diet; M, ApoE^– /–^ +high-fat diet; M+Rosu, ApoE^– /–^ +high-fat diet+Rosu.

Next, we set out to verify the changes of the Pyk2/MCU pathway during AS. To investigate whether the Pyk2/MCU pathway was associated with AS, we examined the related proteins Pyk2, MCU, and caspase-3 by western blot. The results showed that the above-mentioned three proteins were enhanced obviously in the model group compared with the control group, respectively (Figures 3A–D). In addition, in the CH group (C57BL/6J+high-fat diet), the expression of the three proteins revealed upregulation, especially caspase-3 which increased significantly, which together supported that the relevant indicators were activated with the severity of the disease. Moreover, with the treatment of rosuvastatin, these related proteins decreased significantly.

**FIGURE 4:**
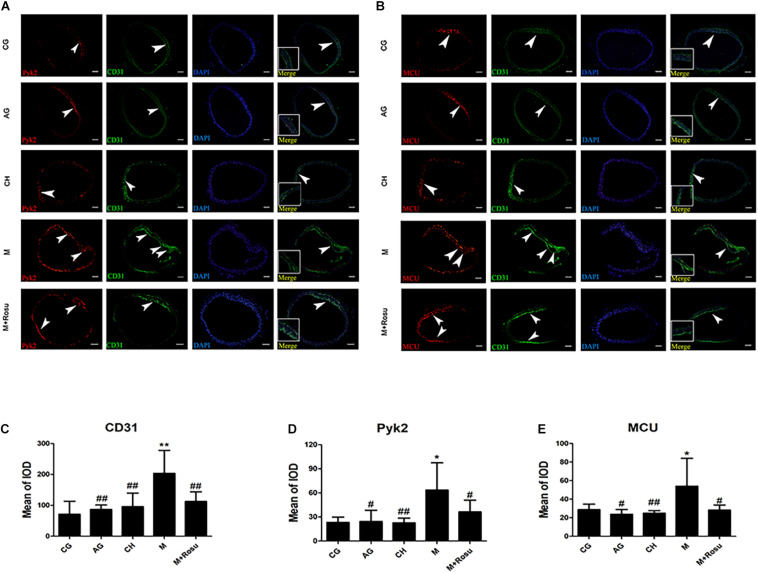
The expression levels of Pyk2/mitochondrial calcium uniporter (MCU) after atherosclerosis in different groups by immunofluorescence. **(A**,**B)** Tissue immunofluorescence; arrowhead: the prominent position of the lesion in the intima. Scale bar: 100 μm. **(C–E)** Mean of integrated option density of Pyk2, MCU, and caspase-3. Data are shown as mean ± SD of at least three independent experiments. *P < 0.05, **P < 0.01 *versus* vehicle control (CG); ^#^*P* < 0.05, ^##^*P* < 0.01 versus vehicle model (M); one-way ANOVA and Mann–Whitney nonparametric tests were applied to compare variables between two groups (*a* = 0.05 as inspection level). CG, C57BL/6J+general diet; AG, ApoE^– /–^ +general diet; CH, C57BL/6J+high-fat diet; M, ApoE^– /–^ +high-fat diet; M+Rosu, ApoE^– /–^ +high-fat diet+Rosu.

We further verified the expression of Pyk2 and MCU in the endothelial layer of the arterial wall that was subjected to AS by immunofluorescence (Figures 4A–E). As expected, both MCU and Pyk2 were obviously expressed in the model group compared to other groups, which was consistent with the western blot data. At the same time, we used CD31 to locate the arterial wall endodermis, which revealed the increased expression of CD31 in the thickened artery intima. CD31 was significantly increased in the model group, while the Pyk2, MCU, and CD31 protein levels showed a downward trend after rosuvastatin intervention. Based on the above-mentioned results, we considered that the Pyk2/MCU pathway activity is upregulated during atherosclerosis and suppressed by rosuvastatin at some levels.

### The Pathological and Ultrastructural Changes of HUVECs Induced by H_2_O_2_ Insults

**FIGURE 5:**
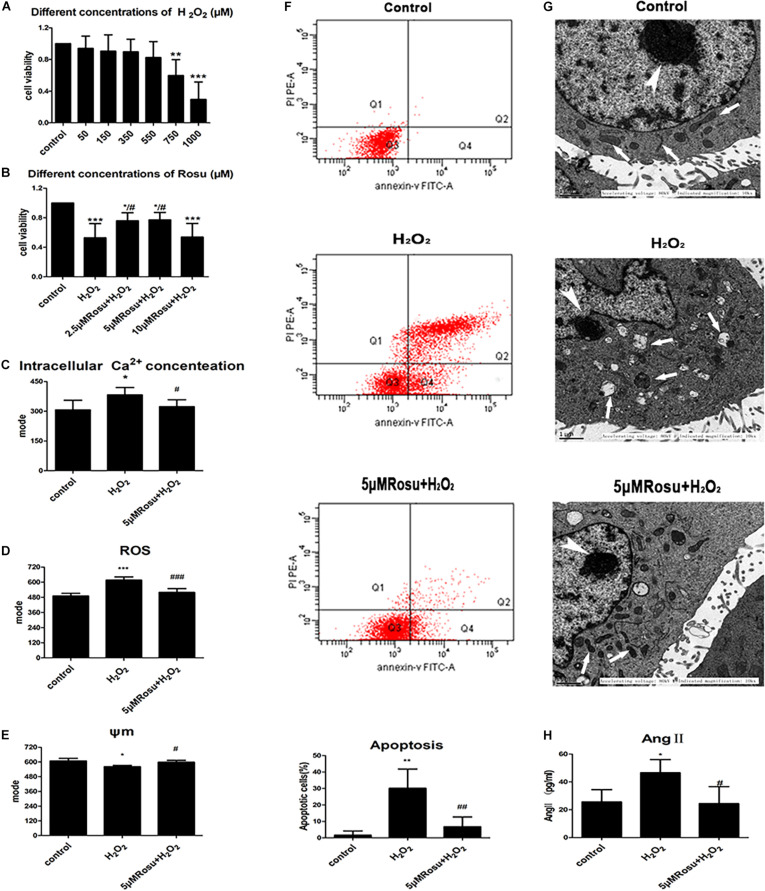
Rosuvastatin intervention promotes mitochondrial function and reduces apoptotic cells from H_2_O_2_-induced human umbilical vein endothelial cells (HUVECs) injury. **(A)** HUVECs viability was reduced when exposed to H_2_O_2_ (750/1,000 μM, 24 h). **(B)** Rosuvastatin (pretreatment 2 h, 2.5/5 μM) protected cells from H_2_O_2_-induced injury in HUVECs. **(C–F)** H_2_O_2_-induced endothelial injury caused an increase in intracellular free Ca^2+^ **(C)**, and the release of ROS **(D)**, reduction of ΔΨm **(E)**, apoptotic cell augmentation **(F)**, and mitochondrial damage when compared to the control group. Rosuvastatin pretreatment was able to reduce the number of apoptotic cells, maintain ΔΨm, reduce the store of intracellular free Ca^2+^ as well as ROS production and protect against H_2_O_2_-induced endothelial injury simultaneously. **(G)** Ultrastructural changes of endothelial cells in different groups. A structured endothelial cell with normal morphology in the control group, including an evident nucleolus (thick arrow) and an intact nuclear membrane, cytoplasmic mitochondria (thin arrow), and endoplasmic reticulum (thin arrow). Dilated mitochondria (thin arrow), many folds in the nuclear membrane, and the exposed nucleolus (thick arrow) are shown in the H_2_O_2_-induced injury group. Through rosuvastatin pretreating, the cell morphology seemed to be recovering, with the nucleolus less damaged (thick arrow), the nuclear membrane damage being relatively minor, and with reduction in mitochondrial swelling (thin arrow). **(H)** The concentration of Ang II in different cell groups. Data are shown as mean ± SD of at least three independent experiments. The significance of the four quadrants represented in the picture of apoptosis: Q1 represents the necrotic cells, Q2 represents the late apoptotic cells, Q3 represents the normal cells, and Q4 represents the early apoptotic cells. We counted the total number of Q2+Q4 as the apoptotic cells. *P < 0.05, **P < 0.01, ***P < 0.001 *versus* vehicle control; ^#^*P* < 0.05, ^##^*P* < 0.01, ^###^*P* < 0.001 *versus* vehicle H_2_O_2_ group, one-way ANOVA.

In order to further study the pathological changes related to atherosclerosis, we used HUVECs to establish a H_2_O_2_-induced endothelial cell injury model. MTS was used to further examine the HUVECs’ viability after H_2_O_2_-induced injury. The results showed that 750 and 1,000 μM H_2_O_2_ significantly reduced the cell viability after 24 h (Figure 5A). In addition, the protective effect of rosuvastatin on cell viability increased when the concentration of rosuvastatin was 2.5 and 5 μM. However, it would not show a significantly protective effect compared with the control group when the concentration of rosuvastatin was 10 μM (Figure 5B). We also found that H_2_O_2_ stimulation increased intracellular Ca^2+^ and intracellular ROS and reduced the mitochondrial membrane potential, while rosuvastatin reversed the changes induced by H_2_O_2_ (Figures 5C–E). In addition, the data revealed a significant increase in the apoptotic rates in the H_2_O_2_ group compared with the control group. H_2_O_2_-induced apoptosis of HUVECs was also inhibited by rosuvastatin (Figure 5F).

Furthermore, we observed morphological changes in the ultrastructure of endothelial cells exposed to different treatments. The endothelial cells in the control group had no edema, the nucleus and nucleoli were obvious, the nuclear membrane was complete, and the cytoplasmic mitochondria, endoplasmic reticulum, and other organelles were complete and clearly visible (Figure 5G). In the H_2_O_2_ group, the endothelial cells appeared to undergo cytoplasmic condensation, the mitochondria were swollen, and the mitochondrial crest disappeared. Furthermore, vacuolization was observed, and the endoplasmic reticulum and nuclear membrane were dissolved. When the cells were pretreated with rosuvastatin, the damage was significantly reduced compared with the H_2_O_2_ group. The nucleoli were clearer than those of the model group, and the nuclear membranes, mitochondria, endoplasmic reticulum as well as other organelles presented less damage, which showed its protective effect on H_2_O_2_-induced endothelial cell injury.

We further examined the damage mechanisms of endothelial cells exposed to H_2_O_2_ (Figure 5H). The results showed that H_2_O_2_ significantly increased Ang II levels, while rosuvastatin inhibited Ang II elevation induced by H_2_O_2_. A previous study had reported that the calcium-dependent activation of tyrosine kinase Pyk2 could be induced by Ang II in vascular endothelial cells (Tang et al., 2000; Satoh et al., 2001). Our results demonstrated that endothelial cell injury was accompanied by an increase in Ang II in the *in vitro* model and took the findings of Tang et al. (2000); Satoh et al. (2001), and Nakashima et al. (2006) into consideration, suggesting that this might provide an interesting evidence between Ang II and Pyk2/MCU signaling. In conclusion, we observed the changes of intracellular free Ca^2+^ (Figure 5C), ROS (Figure 5D), the change of mitochondrial membrane potential (Figure 5E), and cell apoptosis (Figure 5F) in HUVECs exposed to H_2_O_2_ by flow cytometry. These results indicate that rosuvastatin may promote its protective effect through which it reduces Ca^2+^ overload, ROS generation, and the release of apoptotic proteins and improves mitochondrial membrane potential. Whether or not the specific functions of the above-mentioned descriptions are related to the Pyk2/MCU pathway requires to be further identified.

### Inhibition of the Pyk2/MCU Pathway by Rosuvastatin Protects Endothelial Cells From H_2_O_2_-Induced Injury

**FIGURE 6:**
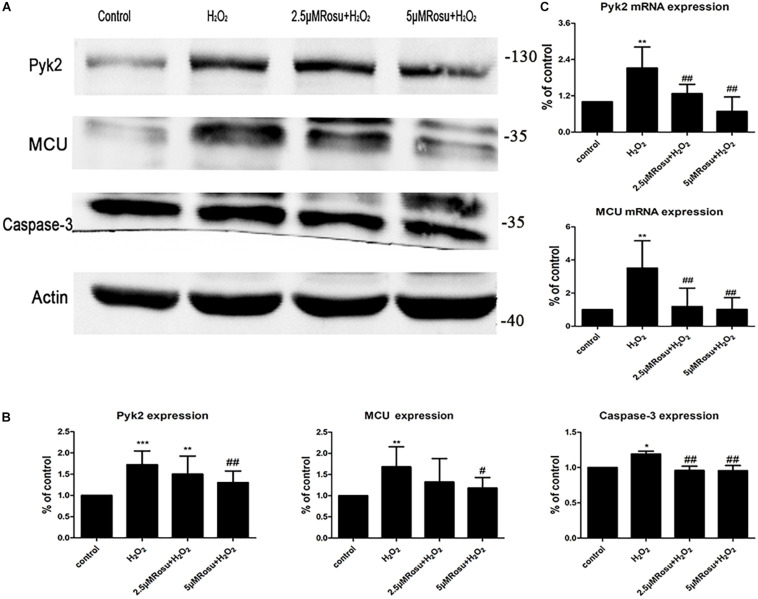
The change of Pyk2/mitochondrial calcium uniporter (MCU) and caspase-3 protein expression in H_2_O_2_-induced human umbilical vein endothelial cells (HUVECs) injury. **(A)** Protein expression of Pyk2/MCU and caspase-3 in different groups. **(B)** Statistical graph of panel **(A)**. **(C)** The level of Pyk2 and MCU mRNA in H_2_O_2_-induced HUVEC injury. Data are shown as mean ± SD of at least three independent experiments. *P < 0.05, **P < 0.01, ***P < 0.001 *versus* vehicle control; ^#^*P* < 0.05, ^##^*P* < 0.01 *versus* vehicle H_2_O_2_ group, one-way ANOVA.

In order to further examine whether the Pyk2/MCU pathway is a target for reversing atherosclerosis, we investigated the changes in Pyk2 and MCU at the transcript level and protein level on cell models (Figure 6). The changes of mRNA expression in Pyk2 and MCU were detected by using real-time quantitative PCR at different treatment groups. As expected, there was a marked increase in MCU mRNA expression after H_2_O_2_-induced injury relative to the control group, which was consistent with the protein expression results (Figures 6A,B). In addition, we observed an increase in Pyk2 mRNA expression in H_2_O_2_-induced HUVEC injury (Figure 6C), and this difference was statistically significant when compared to the control group. On the contrary, the cells pretreated with rosuvastatin for 2 h showed a significant decrease in Pyk2 and MCU mRNA expression compared to the H_2_O_2_ group, which was in agreement with our western blot results.

On the other hand, the expression of both MCU and Pyk2 proteins increased significantly in H_2_O_2_ group than the control and reduced in the high-dose rosuvastatin group compared to the H_2_O_2_ group (Figures 6A,B). In addition, we also measured the expression level of caspase-3 (Figures 6A,B), and there is a small increase after H_2_O_2_ treatment when compared to the control group (We used unpaired Student’s *t*-test to confirm that caspase-3 is upregulated with the H_2_O_2_ intervention. *n* = 5, *P < 0.05, control* vs. *H_2_O_2_ group*). We also found that two rosuvastatin groups reduced caspase-3 protein significantly. Thus, we concluded again that the Pyk2/MCU pathway may be involved in H_2_O_2_-induced endothelial cell injury. Rosuvastatin may inhibit the Pyk2/MCU pathway and endothelial cell apoptosis, which protected the endothelial cells from oxidative stress caused by H_2_O_2_.

### Down-Regulation of Pyk2 by shRNA on H_2_O_2_-Induced HUVEC Injury

**FIGURE 7:**
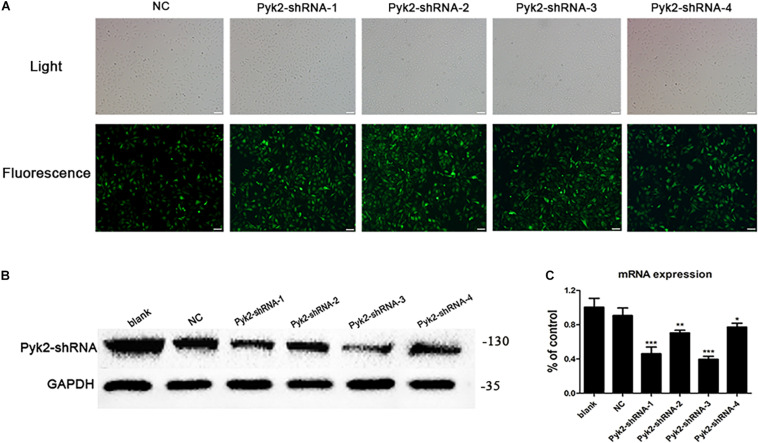
Pyk2-shRNA lentivirus transfection efficiency observation of human umbilical vein endothelial cells (HUVECs). **(A)** Fluorescence results of HUVECs infected with different targeting sequences of Pyk2 gene (×200). Scale bar: 100 μm; target sequence (NC: GCTTCGCGCCGTAGTCTTA; Pyk2-shRNA-1: GCTACTTGCCAGAAGACTTCA; Pyk2-shRNA-2: GCAGCATAGAGTCAGACATCT; Pyk2-shRNA-3: GCTGTACTCACTGCAGATATG; Pyk2-shRNA-4: GCGGCAAATCCTGGACAAACA). **(B)** Protein expression results of HUVECs infected by different Pyk2-shRNA lentivirus. **(C)** mRNA expression results of HUVECs infected by different Pyk2-shRNA lentivirus. Data are shown as mean ± SD of at least three independent experiments. *P < 0.05, **P < 0.01, ***P < 0.001 *versus* NC group, one-way ANOVA.

**FIGURE 8:**
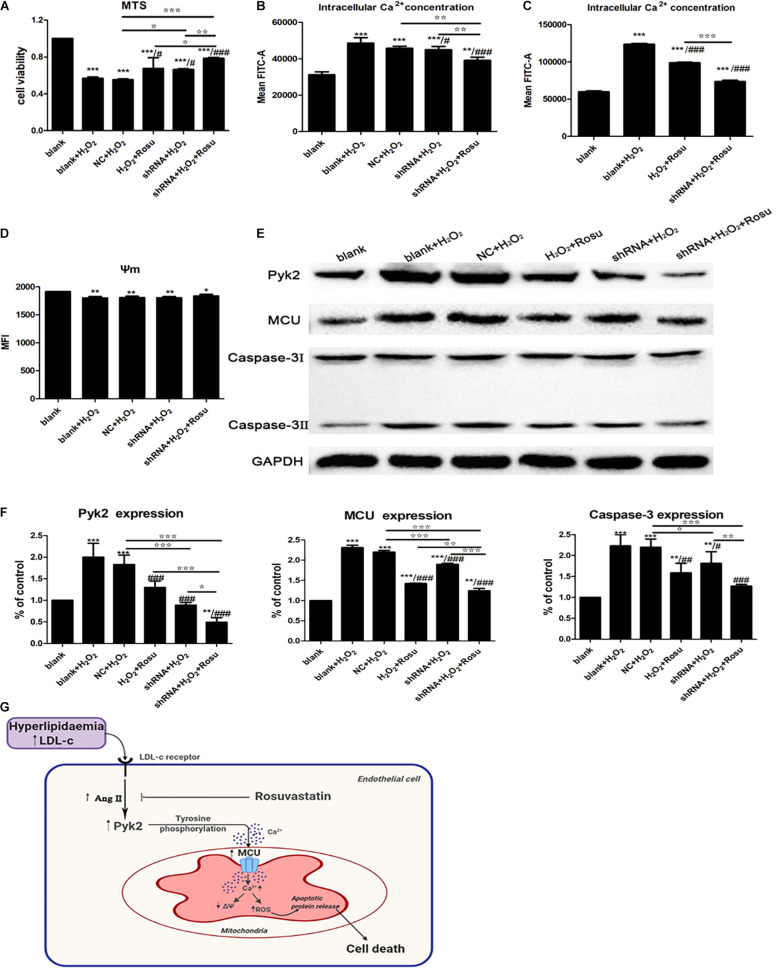
The effect of Pyk2 down-regulation on H_2_O_2_-induced human umbilical vein endothelial cells (HUVECs) injury and regulation of the Pyk2/mitochondrial calcium uniporter (MCU) pathway in endothelial cell. **(A)** Detection of cell viability by 3-(4,5-dimethylthiazol-2-yl)-5-(3-carboxymethoxyphenyl)-2-(4-sulfophenyl)- 2H-tetrazolium assay after transfection with shRNA. **(B–D)** Detection of intracellular free Ca^2+^ and mitochondrial membrane potential in different groups by flow cytometry. **(E)** Pyk2, MCU, and caspase-3 protein expression in different groups by western blot. **(F)** Statistical graph of panel **(E)**. Data are shown as mean ± SD of at least three independent experiments. *P < 0.05, **P < 0.01, ***P < 0.001 *versus* vehicle blank group (control); ^#^*P* < 0.05, ^##^*P* < 0.01, ^###^*P* < 0.001 *versus* vehicle H_2_O_2_ group (blank+H_2_O_2_); ✩*P* < 0.05, ✩✩*P* < 0.01, ✩✩✩*P* < 0.001, NC+H_2_O_2_ vs. shRNA+H_2_O_2_, NC+H_2_O_2_ vs. shRNA+H_2_O_2_+Rosu; H_2_O_2_+Rosu vs. shRNA+H_2_O_2_+Rosu, shRNA+H_2_O_2_ vs. shRNA+H_2_O_2_+Rosu, one-way ANOVA. **(G)** Endothelial angiotensin II involvement-Pyk2-dependent MCU phosphorylation initiates mitochondrial Ca^2+^ entry, which, in turn, induces reactive oxygen species generation and a decrease in mitochondrial membrane potential (MMP/ΔΨ) and eventually leading to cell death.

In order to verify the effect of Pyk2 down-regulation on H_2_O_2_-induced HUVEC injury, we conducted the lentivirus transfection (MOI = 50) and used rt-qPCR and western blot technologies to check the interference efficiency of different viruses (Pyk2-shRNA-1, Pyk2-shRNA-2, Pyk2-shRNA-3, and Pyk2-shRNA-4) targeting sequences (Figures 7A–C). Based on the results, the Pyk2-shRNA-3 lentivirus was selected for subsequent formal experiments. To gain insights into the underlying mechanisms regarding whether the Pyk2/MCU pathway was associated with endothelial cell injury, a shRNA technique was used in the next experiments. This section is divided into six groups: blank group, H_2_O_2_ group (blank+H_2_O_2_), NC group (NC+H_2_O_2_), rosuvastatin group (H_2_O_2_+Rosu), shRNA group (shRNA+H_2_O_2_), and (shRNA+H_2_O_2_+Rosu) group. Then, we made a pairwise comparison based on the statistical analysis results, which contain blank+H_2_O_2_ vs. shRNA+H_2_O_2_, blank+H_2_O_2_ vs. H_2_O_2_+Rosu, blank+H_2_O_2_ vs. NC+H_2_O_2_, NC+H_2_O_2_ vs. shRNA+H_2_O_2_, shRNA+H_2_O_2_ vs. shRNA+H_2_O_2_+Rosu, and H_2_O_2_+Rosu vs. shRNA+H_2_O_2_+Rosu. The results revealed that, compared with the H_2_O_2_ group (blank+H_2_O_2_), cell viability was significantly higher in the shRNA group (shRNA+H_2_O_2_), and rosuvastatin group (H_2_O_2_+Rosu) can also promote cell viability. Cell viability in the NC group (NC+H_2_O_2_) showed no significant changes when compared with the H_2_O_2_ group (blank+H_2_O_2_). A comparison of cell survival between the NC group (NC+H_2_O_2_) and shRNA group (shRNA+H_2_O_2_) revealed dramatic differences. In addition, both giving rosuvastatin pretreatment and Pyk2 of low expression (shRNA+H_2_O_2_+Rosu) showed a stronger protective effect than using rosuvastatin solely (Figure 8A). The results directly indicated that inhibition of Pyk2 can prevent H_2_O_2_-induced endothelial cell injury, while rosuvastatin had a protective role which degenerated Pyk2 expression, consistent with our above-mentioned observations. The changes in mitochondrial membrane potential and intracellular free Ca^2+^ concentration were detected by flow cytometry. We observed a downward trend in the concentration of intracellular free Ca^2+^ in the transfected shRNA group (shRNA+H_2_O_2_), although no statistical significance was found compared to the H_2_O_2_ group (blank+H_2_O_2_). The NC group (NC+H_2_O_2_) showed no significant changes when compared with the shRNA group (shRNA+H_2_O_2_) in intracellular free Ca^2+^. However, after treatment with rosuvastatin, there was a significant decrease in intracellular Ca^2+^, which was even more obvious when the gene of Pyk2 was of low expression simultaneously. Both giving rosuvastatin pretreatment and Pyk2-shRNA reduced intracellular free Ca^2+^ better than rosuvastatin alone or merely Pyk2 of low expression with shRNA technique (Figures 8B,C). In the measurement of MMP, we found that the trend was slightly obvious when intervened with rosuvastatin, although this difference was not statistically significant (Figure 8D and Table 1). Accordingly, it appears that inhibition of Pyk2 expression may reduce Ca^2+^ influx, and rosuvastatin can also reverse the injury caused by H_2_O_2_ stimulation through this pathway.

**TABLE 1:** Mean fluorescence intensity (MFI) of mitochondrial membrane potential in different groups.

Group	Blank	Blank+H_2_O_2_	NC+H_2_O_2_	shRNA+H_2_O_2_	shRNA+H_2_O_2_+Rosu
MFI	1,914.57 ± 1	1,801.73 ± 26.49**	1,809.63 ± 22.49**	1,805.53 ± 22.06**	1,835.37 ± 30.96*

*P < 0.05, **P < 0.01 vs. blank group (mean ± SD; *n* = 3), one-way ANOVA.

We further evaluated the proteins’ expression of Pyk2, MCU, and caspase-3 by western blot, which showed that the expression of these proteins in the shRNA group (shRNA+H_2_O_2_) was notably lower compared with those in the H_2_O_2_ group, implying that H_2_O_2_ activated the Pyk2/MCU pathway, and apoptotic protein release was prohibited by Pyk2 shRNA (Figures 8E,F). There were marked changes of these proteins’ expression in the shRNA group (shRNA+H_2_O_2_) compared with the NC group (NC+H_2_O_2_). What we can see in the data is a significant decrease in the expression of the three proteins when rosuvastatin was administered, thus suggesting that the Pyk2/MCU pathway may be the target of rosuvastatin, and the drug protects endothelial cells from H_2_O_2_ insult through inhibiting the signaling. Meanwhile, treatment with Pyk2 shRNA and rosuvastatin at the same time showed a stronger inhibition of Pyk2, MCU, and caspase-3 (Figures 8E,F). Collectively, these observations indicate that inhibition of the Pyk2/MCU pathway by shRNA technique and/or rosuvastatin intervention eliminates the excessive apoptotic cells produced, mitochondrial injury, and Ca^2+^ accumulated in the endothelial cells, thus reducing the occurrence of injury of endothelial cells caused by mitochondrial damage and improving the cells’ viability.

## Discussion

Atherosclerosis is associated with a number of life-threatening cardiovascular disorders. The exact mechanisms of action that underlie these associations remain unclear (Lusis, 2000; Lozano et al., 2012). Identification of specific molecules that mediate resistance to AS may contribute to facilitate the development of novel therapies and improve response to currently available therapies. In this study, we identified the Pyk2/MCU pathway as a new regulator for atherosclerosis. Briefly, Pyk2/MCU regulates mitochondrial Ca^2+^ uptake, ROS production, mitochondrial membrane potential, and apoptotic signaling, which, in turn, affects the occurrence and development of atherosclerosis. On the other hand, we discovered that rosuvastatin, an important lipid-lowering treatment drug, may affect the Pyk2/MCU pathway, thus preventing the development of AS (Figure 8G). This model was proven *in vivo*, by using mice exposed to high-fat diet, and *in vitro*, using a H_2_O_2_-induced endothelial cell injury model. Notably, these results provide new insights into the molecular basis of the Pyk2/MCU pathway. The inhibition of the Pyk2/MCU pathway which further modulates mitochondrial Ca^2+^ handling and apoptosis in atherosclerosis may confer a protective effect to the arteries against atherosclerosis.

### The Regulation of the Pyk2/MCU Pathway Underlying Endothelial Cell Injury

Many factors are involved in the pathophysiological process of atherosclerosis. Although the molecular details underlying atherosclerotic lesion development have not yet been elucidated, endothelial dysfunction has been recognized as an integral component in the initiation and progression of atherosclerosis (Sun et al., 2011). The role of oxidative stress in the development of endothelial dysfunction and subsequent atherosclerotic disease has been an area of interest over the few decades. Initial work in this field considered ROS as the primarily toxic metabolic by-product with adverse effects on vascular function from direct damage to key cellular proteins (Widlansky and Gutterman, 2011). Mitochondria, as a major cellular source of ROS, have recently garnered increased attention for their contribution to the detrimental effects of cardiovascular and cerebrovascular disease (Walters et al., 2016). The increase of ROS production from mitochondria has been closely associated with excessive mitochondrial Ca^2+^ uptake (O-Uchi et al., 2014). Furthermore, mitochondrial Ca^2+^ overload results in mitochondrial membrane potential depolarization that opens a mitochondria permeability transition pore, generates intracellular ROS, and finally releases the proapoptotic proteins into the cytosol, which, in turn, results in cell injury and death (Zhang et al., 2018). Thus, the potential risk of mitochondrial Ca^2+^ overload leading to apoptosis and the signaling of mitochondrial Ca^2+^ uptake in endothelial cells need to be precisely regulated. In this experiment, we successfully established an EC injury model with H_2_O_2_ and found that H_2_O_2_ caused a decrease in cell viability, an increase in ROS production, and a destruction of mitochondrial function. We found similar trends in the ApoE^–/–^ mouse model of atherosclerosis. To sum up, studies are consistent, and we believe that oxidative stress and mitochondrial damage are involved in atherosclerotic endothelial damage and accelerate the process of atherosclerosis.

Recently, it has been discovered that a highly negative membrane potential (ΔΨ) regulates the Ca^2+^ that enters the mitochondria through a selective inward rectifying MCU channel (Hajnoczky et al., 2006). The channel, which is composed of mitochondrial matrix resident proteins MICUs, MCUb, MCUR1, and EMRE (Kamer and Mootha, 2014; Lee et al., 2015), is transcriptionally regulated by upstream Ca^2+^ cascade, post-translational modification, and divalent cations. The mode of regulation can either inhibit or enhance MCU channel activity and thus regulate mitochondrial metabolism and cell fate. Under conditions of oxidative stress, C97 at the structure of N-terminal domain of MCU gets *S*-glutathionylated, promoting MCU oligomerization and increasing mitochondria Ca^2+^ uptake, leading to Ca^2+^ overload and swelling of the mitochondria (Nemani et al., 2018). Therefore, mitochondria damage has an important role in atherosclerosis, and calcium imbalance is one of the significant links. However, the precise regulation and molecular mechanism behind this process is still unclear. In this study, we found that an increase in MCU protein is accompanied with the reduction of mitochondrial membrane potential, production of ROS, cell apoptosis induction, and mitochondria swelling in the H_2_O_2_-induced endothelial cell injury and AS mouse model, while inhibition of MCU reversed those processes. Therefore, we consider that MCU participates in the pathological process of atherosclerosis.

Pyk2 is a 116-kDa cytoplasmic tyrosine kinase which is a member of the focal adhesion kinase family (Zhang et al., 2018). Previous studies have demonstrated that activation of Pyk2 in PVECs regulates Ca^2+^ dynamics, which may be responsible for EC death (Satoh et al., 2001). In this study, we want to confirm whether Pyk2 is stimulated by other factors and the relationship between Pyk2 and MCU in the process of atherosclerosis. The existing evidence had clearly shown that Pyk2 modulates mitochondrial calcium uptake through phosphorylation of the MCU in cardiomyocyte (O-Uchi et al., 2014), and the Pyk2/MCU pathway is activated in a rat cerebral ischemia model, which is responsible for mitochondrial dysfunction and neuronal apoptosis (Zhang et al., 2018). Interestingly, a separate study confirmed the calcium dependence of tyrosine kinase Pyk2 can be activated by Ang II in PVECs (Satoh et al., 2001). The role of Pyk2 in the regulation of Ang II-induced signaling pathways was certified in mediating VSMC growth, which was later reported (Rocic et al., 2001). Particularly, Ang II has a crucial role in several vascular pathologies including aortic aneurysm and atherosclerosis (Daugherty et al., 2000, 2010), and Ang II-induced vascular hypertrophy is mediated by intracellularly produced ROS (Nakashima et al., 2006). Meanwhile, by the involvement of Ang II, ROS can mediate the Ca^2+^-dependent transactivation of epidermal growth factor receptor (Yin et al., 2003). It can be seen that, under some pathological conditions, Ang II and ROS will interact with each other, which are related to the calcium dependence of tyrosine kinase Pyk2. Studies have also shown that Ang II infusion stimulates the progression of atherosclerosis in ApoE^–/–^mice (Weiss et al., 2001; Rateri et al., 2011). In addition, Ang II cross-talks with several tyrosine kinases *via* AT_1_Rs, including receptor tyrosine kinases (EGFR, PDGF, insulin receptor and nonreceptor tyrosine kinases, c-Src family kinases, Ca^2+^-dependent Pyk2, focal adhesion kinase, and Janus kinases) (Mehta and Griendling, 2007). Given the relationship described above, we then investigated whether the Pyk2/MCU pathway is correlated with the pathological process of AS and Ang II could influence this pathway. In the present study, it is possible that the activation of Pyk2 is connected with the Ang II increase since we observed that the Pyk2/MCU pathway is activated, and Ang II was upregulated simultaneously in the H_2_O_2_-induced EC injury model. This is in line with evidence suggesting that Ang II stimulated a calcium-sensitive tyrosine kinase Pyk2 in PVECs (Tang et al., 2000; Satoh et al., 2001). However, we cannot see the obvious change of Ang II in the atherosclerosis mouse model in spite of it being inhibited by the treatment of rosuvastatin, which is a limitation of our study. Therefore, it becomes our next goal which requires to be further demonstrated because we want to completely reflect the *in vivo* situation and we do not stop in the future. Thereafter, we used shRNA technique to reduce Pyk2 expression and interestingly discovered that MCU expression decreased, the mitochondria were protected, and the apoptosis was reduced, which, in turn, led to an alleviation of H_2_O_2_-induced EC injury. Therefore, we consider that the activation of the Pyk2/MCU pathway has a crucial role in the EC injury process and may be involved in the pathological process of atherosclerosis. Next, further verification would address the question whether inhibition of the Pyk2/MCU pathway is likely to be a new therapeutic target in the animal model for preventing the development of atherosclerosis.

### Pyk2/MCU Pathway May Be a New Target of Reversing Atherosclerosis

Treatments for atherosclerosis may include healthy lifestyle changes and medicines, such as oral lipid-lowering drugs (Grundy et al., 2019). However, there is no cure for atherosclerosis so far. Statin is important for effective inhibition of cholesterol synthesis (Vale et al., 2014), improving physiological function of vascular endothelium (Duran et al., 2010), anti-inflammatory reaction (Naito et al., 2006), decreasing vascular plaque area (Takayama et al., 2016), anti-apoptosis effect (Geng et al., 2019), anti-oxidant stress, protecting mitochondrial function (Liu et al., 2017), and so on. This study confirmed that rosuvastatin can inhibit the Pyk2/MCU pathway during atherosclerosis process in ApoE^–/–^ mice with high-fat diet and HUVECs exposed to H_2_O_2_, further promoting mitochondrial function by maintaining the MMP, blocking intracellular Ca^2+^ influx, and reducing ROS release. These results suggest that the Pyk2/MCU pathway may serve as a new target for atherosclerosis. The function of rosuvastatin on the AS mouse model was consistent with the effect of the Pyk2-shRNA results in HUVECs, and that drug declined the activation of Pyk2/MCU pathway as well as protected mitochondrial function, which further confirmed that rosuvastatin protected ECs by inhibiting the Pyk2/MCU pathway. However, how do statins inhibit the Pyk2/MCU pathway? Through consulting the literature, we found that atherosclerosis or endothelial injury caused higher Ang II, and statins can inhibit Ang II (Colucci et al., 2013). In our experiment, the results showed that, in the H_2_O_2_-induced EC injury model, there was Ang II increase, and it may be correlated to the Pyk2/MCU pathway, while rosuvastatin decreased Ang II in both *in vitro* and *in vivo* situations. Although there are few experimental results on the relationship between Ang II and the Pyk2/MCU pathway in our study at present, the present results provide more possibilities for our future research. These data revealed the underlying mechanism of the EC protection conferred by rosuvastatin during atherosclerosis, which may be explained by the inhibition of Ang II involved in the Pyk2/MCU pathway activation, then decreasing intracellular ROS production and Ca^2+^ influx, protecting the mitochondrial function and ultimately preventing EC apoptosis (Figure 8G).

## Conclusion

This study is the first to report the association between the Pyk2/MCU pathway and atherosclerosis. We provide a body of evidence demonstrating that Pyk2/MCU may be a new target for inhibiting or reversing atherosclerosis. However, additional studies are required to elaborate the specific molecular mechanisms for this effect and eventually find new molecules which target on this pathway.

## Data Availability Statement

The raw data supporting the conclusions of this article will be made available by the authors, without undue reservation.

## Ethics Statement

The animal study was reviewed and approved by Hebei Medical University institutional animal care.

## Author Contributions

XL and PF designed the experiments. YzZ performed and wrote the manuscript. XY, ZL, KB, TL, ZM, BW, LM, HL, KZ, LjL, and YyZ helped with experiments. YpZ, JQ, LL, SL, and JC contributed to the discussion and review of the manuscript. XL obtained the funding, designed the experiments, and modified the manuscript. All authors have read and approved the final manuscript.

## Conflict of Interest

The authors declare that the research was conducted in the absence of any commercial or financial relationships that could be construed as a potential conflict of interest.
